# Mitochondrial genome of *Chrysochares punctatus* (Coleoptera: Chrysomelidae: Eumolpinae) and phylogenetic analysis

**DOI:** 10.1080/23802359.2020.1711823

**Published:** 2020-01-16

**Authors:** Run-Qiu Feng, Li-Jun Zhang, Min Li, Jia Liu, Chun-Li Wen, Ming-Long Yuan

**Affiliations:** Key Laboratory of Grassland Livestock Industry Innovation, Ministry of Agriculture and Rural Affairs, State Key Laboratory of Grassland Agro-Ecosystems, College of Pastoral Agricultural Science and Technology, Lanzhou University, Lanzhou, People's Republic of China

**Keywords:** Insects, leaf beetles, mitochondrial DNA, phylogeny

## Abstract

Here, we determined the nearly complete mitochondrial genome (mitogenome) of *Chrysochares punctatus* (Coleoptera: Chrysomelidae: Eumolpinae), an important insect pest on *Apocynum venetum* in Northwestern China. This mitogenome was 14,451 bp long, encoding 13 protein-coding genes (PCGs), 21 transfer RNA genes (tRNAs), and 2 ribosomal RNA genes. The *C. punctatus* mitogenome presented an A + T content of 75.11%, with a positive AT-skew (0.064) and a negative GC-skew (−0.192). Ten PCGs started with a typical ATN codon, whereas the remaining three PCGs started with AAC (*cox1*) and TTG (*nad1* and *nad2*). All tRNAs had a typical secondary cloverleaf structure, except for *trnS1* which lacked the dihydrouridine arm. Bayesian phylogenetic analysis based on the nucleotide sequences of 13 PCGs recovered a phylogeny within Chrysomelidae: (((Chrysomelinae + Galerucinae), (((Eumolpinae, Lamprosomatinae), Cassidinae), Criocerinae)), Bruchinae).

*Chrysochares punctatus* (Coleoptera: Chrysomelidae: Eumolpinae) is an important insect pest on *Apocynum venetum* (Gentianales: Apocynaceae). Here, we sequenced and annotated the mitochondrial genome (mitogenome) of *C. punctatus*. Adult specimens were collected from Altay City (87.55°E, 47.71°N), Xinjiang Uygur Autonomous Region, China, in July 2018. All samples (LZUALT45) have been deposited in the State Key Laboratory of Grassland Agro-Ecosystems, College of Pastoral Agricultural Science and Technology, Lanzhou University, Lanzhou, China. The total genomic DNA was extracted from a single specimen (LZUALT45_1) using a DNeasy Tissue Kit (Qiagen). The *C. punctatus* mitogenome was amplified with a set of universal and specific primers, and sequenced in both directions, following the method of our previous study (Yuan et al. 2016).

The nearly complete mitogenome of *C. punctatus* was 14,451 bp long (GenBank accession number MN745103). This mitogenome contained 13 protein-coding genes (PCGs), 21 transfer RNA genes (tRNAs), and 2 ribosomal RNA genes (*rrnL and rrnS*). The order and orientation of the mitochondrial genes were identical to the inferred ancestral arrangement of insects (Boore 1999). Gene overlaps were found at seven gene junctions and involved a total of 27 bp; the longest overlap (8 bp) existed between *trnW* and *trnC.* A total of 29 bp intergenic spacers were present in seven positions, ranging in the size from 1 bp to 17 bp.

The nucleotide composition of the *C. punctatus* mitogenome was biased toward A and T with an A + T content of 75.11% in J-strand. This mitogenome presented a positive AT-skew (0.064) and a negative GC-skew (−0.192), as found in most insect mitogenomes. The *rrnL* was 1265 bp long with an A + T content of 80.24% and the *rrnS* was 744 bp with an A + T content of 77.28%. Among the 13 PCGs, the lowest A + T content was 67.19% in *cox1*, while the highest was 84.31% in *atp8*. Ten PCGs started with a typical ATN codon: two (*cox2*, *nad6*) with ATC, two (*atp8*, *nad5*) with ATT, two (*nad1*, *nad2*) with TTG, five (*atp6*, *cox3*, *nad4, nad4L*, *cob*) with ATG. The remaining two PCGs began with AAC (*cox1*) or ATA (*nad3*). Five PCGs terminated with a complete end codon TAA, whereas the remaining eight terminated with an incomplete stop codon TA or T. All of the 21 tRNAs, ranging from 61 bp (*trnA*) to 71 bp (*trnK*), had a typical cloverleaf structure, except for *trnS1* which lacked the dihydrouridine arm.

The concatenated nucleotide sequences of 13 PCGs from 35 leaf beetles and two outgroups (*Anoplophora glabripennis* and *Batocera lineolata*) from Cerambycidae were used for phylogenetic analysis, using RAxML-HPC2 on the CIPRES Science Gateway 3.3 (Miller et al. 2010). The maximum likelihood tree strongly supported a closer relationship between *C. punctatus* and *Chrysodinopsis* sp. from the same subfamily Eumolpinae (Figure 1). Phylogenetic relationships among subfamilies were recovered as (((Chrysomelinae + Galerucinae), (((Eumolpinae, Lamprosomatinae), Cassidinae), Criocerinae)), Bruchinae).

**Figure 1.**
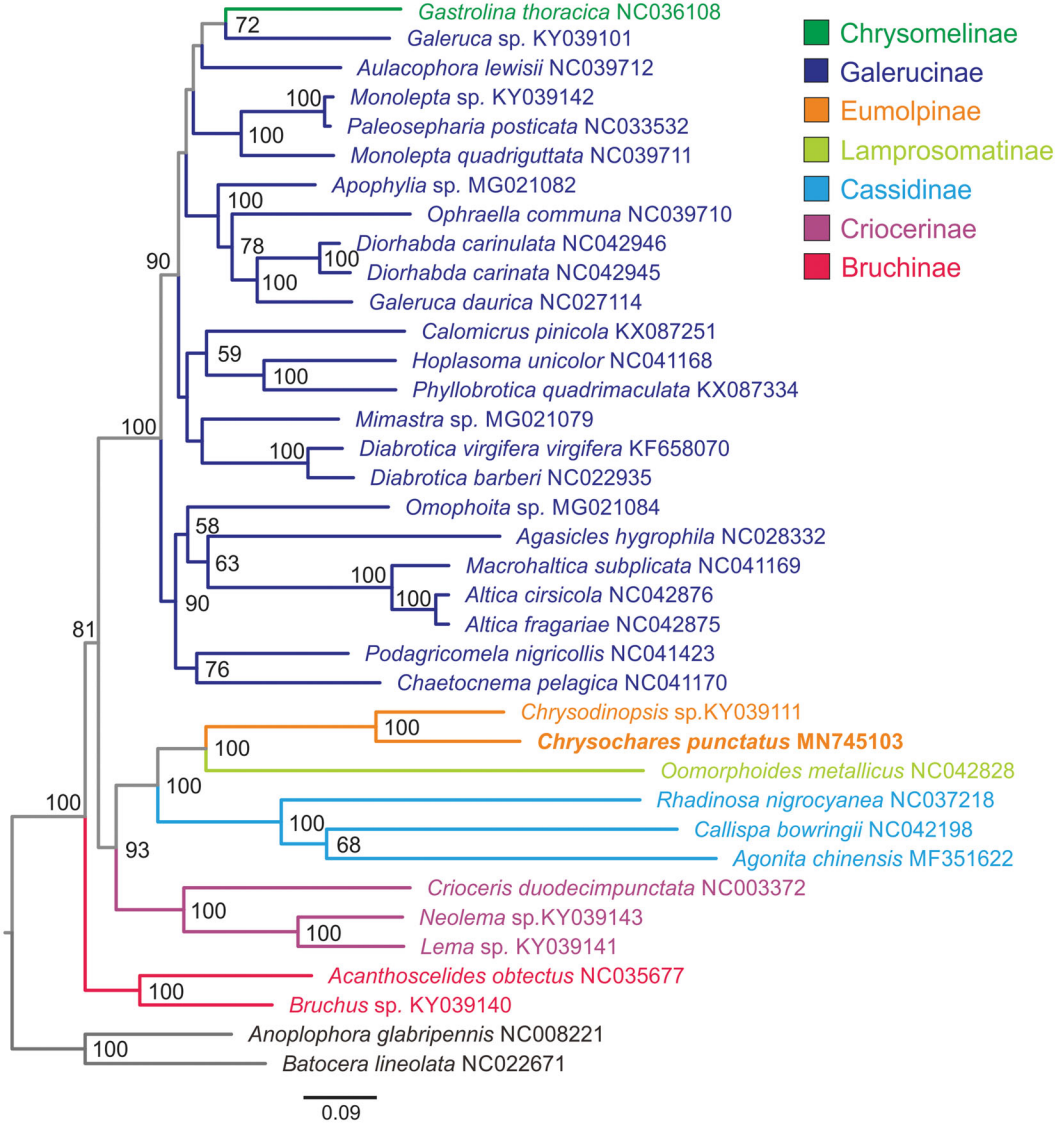
Mitochondrial phylogeny of 35 Chrysomelidae species based on the concatenated nucleotide sequences of 13 mitochondrial protein-coding genes. Bootstrap values less than 50 are not shown on the branch.
